# Human Saliva: Non-Invasive Fluid for Detecting Novel Coronavirus (2019-nCoV)

**DOI:** 10.3390/ijerph17072225

**Published:** 2020-03-26

**Authors:** Zohaib Khurshid, Faris Yahya Ibrahim Asiri, Hamed Al Wadaani

**Affiliations:** 1 Department of Prosthodontics and Dental Implantology, College of Dentistry, King Faisal University, Al-Ahsa 31982, Saudi Arabia; 2 Department of Preventive Dentistry, College of Dentistry, King Faisal University. Al-Ahsa 31982, Saudi Arabia; fasiri@kfu.edu.sa; 3 Department of Surgery, College of Medicine, King Faisal University, Al-Ahsa 31982, Saudi Arabia; hwadani@hotmail.com

**Keywords:** saliva, virus, 2019-nCoV, severe acute respiratory syndrome (SERS-CoV), acute respiratory distress syndrome (ARDS), diagnostics, point-of-care, COVID-2019

## Abstract

The breakthrough of novel coronavirus (2019-nCoV) in Wuhan, a city of China, has damaged the status of health and quality of life. In the sequel of this epidemic or contagious disease, the patient experiences fever, chest paint, chills, a rapid heartbeat, breathing difficulties, pneumonia, and kidney failure. It has been suggested that this disease can spread through human-to-human transmission or by super spreading. By the help of the non-invasive fluid “saliva”, it is easy to detect the virus. This can help with the comfort of the patient as well as healthcare personnel. Under this perspective, we discuss the epidemic situation of 2019-nCOV and its relationship with human saliva.

## 1. Introduction

**Figure 1:**
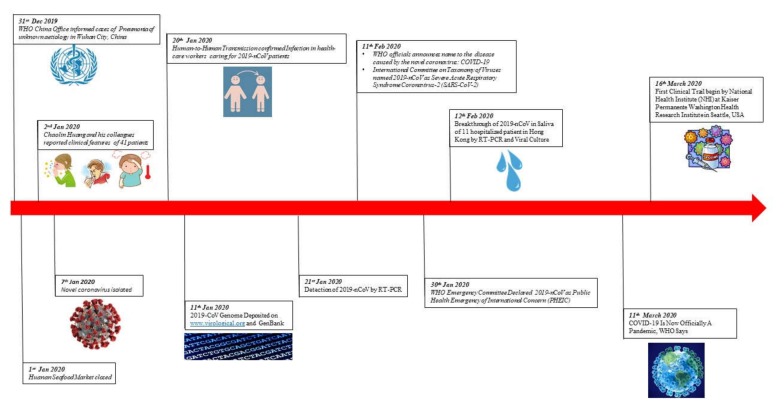
Events related to the 2019-Novel Coronavirus (2019-CoV) outbreak.

In December 2019, patients with pneumonia of unknown cause reported to hospitals in Wuhan, Hubei, China. This unknown cause of pneumonia provoked fear, stress, and panic in China. Within a day, this condition spread to other provinces in China, and health authorities started immediate investigation to characterize and isolated the virus, which, by 7 January 2020, was named the Novel Coronavirus (nCoV) [1]. On 12 January 2020, the World Health Organization (WHO) named this new virus 2019-Novel Coronavirus (2019-nCoV). The infectious disease was named COVID-19 by WHO on 11 February 2020. This virus is part of a diverse family of viruses, consisting of four viral genera (alpha-, beta-, gamma-, and delta-coronaviruses) [2]. They infect different body systems of human and vertebrates like the respiratory, central nervous, hepatic, and gastrointestinal systems [3]. Figure 1 shows the essential events related to the 2019-nCoV outbreak. 

The clinical features of COVID-19, as determined from 99 patients in Wuhan city, China are fever (83%), cough (82%), shortness of breath (31%), muscle pain (11%), confusion (9%), headache (8%), sore throat (5%), rhinorrhea (4%), chest pain (2%), diarrhea (2%), and nausea and vomiting (1%) [4]. A study revealed that reported patients tended to be older males who had the fatal condition of acute respiratory distress syndrome (ARDS) [4]. As of 28 February 2020, the day on which this perspective was finalized, a total of 46 countries other than China have reported to have 2019-nCoV cases (Source WHO Situation Report-38). According to the WHO situation report, globally, 179,000 thousand cases have been confirmed and the number is increasing every hour. Understanding the disease etiology, epidemics, genomics, clinical findings, and treatment options requires extensive data from sampling, laboratory work, and clinical trials [5,6]. Rapid findings can help to control the disease spread as well as further outbreaks of contagious viruses. COVID-2019 transmission occurs person-to-person, either through direct transmission by sneeze, cough, or droplet inhalation, or contact transmission such as ocular contact or through mucous membranes of the eyes and nose and saliva [7,8].

In this perspective, we highlight the hidden capability of saliva for the early detection of any viral, bacterial, or systemic disease [9]. In the past, saliva was proven to be an ideal role for the isolation of proteins, peptides, and sheds of viruses via many molecular assays [10]. The composition of saliva is very informative for analysis or to compare the physiology or pathology of the human body. Currently, salivary biomarkers are helping in the detection of oral cancer, dental caries, periodontal diseases, diabetes, breast cancer, and lung cancer [9,10]. The oral cavity is kept wet by salivary flow, and the normal physiological activities of the oral cavity are maintained by a saliva washout mechanism [9]. A study revealed a large amount of RNA isolation from the saliva of a severe acute respiratory syndrome (SARS)-associated coronavirus patient in National Taiwan University Hospital [11]. In this study, quantitative real-time reverse transcription-polymerase chain reaction (RT-PCR) assay was used to investigate the load of SARS-CoV in the saliva samples. Samples of saliva from 17 patients were confirmed to have lymphopenia, elevated levels of creatine kinase, and thrombocytopenia. On 9 January 2020, WHO published guidelines for the detection of 2019-nCoV using respiratory materials (nasopharyngeal and oropharyngeal swab in ambulatory patients and sputum (if produced) and endotracheal aspirate or bronchoalveolar lavage in patients with more severe respiratory disease) and serum for serological testing (https://www.who.int/publications-detail/laboratory-testing-for-2019-novel-coronavirus-in-suspected-human-cases-20200117 accessed on 28 February 2020). Testing at these sites is painful, uncomfortable, and invasive for the patients, but there is strong evidence for its use in detecting the virus’s presence. A recent paper reported only one case as part of a diagnostic evaluation of sputum from a 2019-nCoV patient, and it was a lower respiratory tract sample [12]. Nasopharyngeal and oropharyngeal swabs are not suitable for monitoring the viral loads compared with saliva samples [11]. 

**Figure 2:**
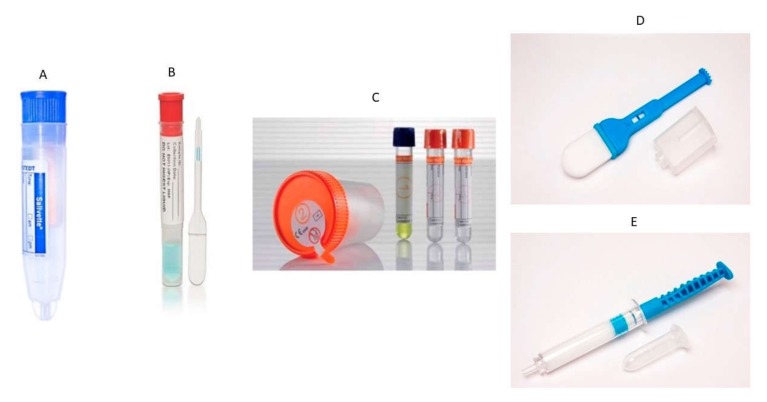
Representation of different saliva collection devices used in the sampling of contagious infectious diseases. (**A**) Salivette^®^ (Sarstedt); (**B**) Quantisal^®^ (Immunalysis); (**C**) SCS^®^ (Greiner-BioOne), (**D**) Versi•SAL^®^, and (**E**) Super•SAL™ by Oasis Diagnostics^®^ Corporation [11,16].

On 12 February 2020, a breakthrough was reported regarding the accuracy of a human saliva sample from eleven COVID-19 patients in a Hong Kong hospital [13]. In this study, consistent detection of coronavirus was reported in the saliva of patients admitted from the first day of hospitalization. The sampling of saliva in this study was done by instructing the patient to cough out saliva from the throat into a sterile container, and this was transported to the laboratory for further analysis. This study demonstrated the advantage of saliva sampling comfortability in an epidemic situation such as COVID-2019 [13]. Further exploring the use of saliva or oral fluid will bring new treatment strategies in the prevention and early detection of COVID-19 [14]. By using saliva as a form of liquid biopsy, healthcare providers, doctors, nurses, and paramedic staff will be safe from the transmission of disease. This method of sampling is advantageous compared with the use of nasopharyngeal aspirates, oropharyngeal swabs, and nasopharyngeal swabs. Previously, it has been reported that the SARS-CoV RNA can be detected from saliva and throat wash [11]. There are many saliva collection devices available in the market for safe and sterile collection without compromising the quality and quantity [15]. Figure 2 shows commercially available saliva sampling devices and their company names, which can be accessed by all researchers, healthcare providers, doctors, microbiologists, and virologists for the handling of samples. Further investigation on salivary biomarkers related to COVID-2019 will open a corridor for optimizing cost-effective point-of-care (POC) technology [16,17]. 

## 2. Conclusions and Future Direction

Further investigation should investigate the diagnostic capability of human saliva for identifying COVID-2019, SARS-COV, MERS, ZIKV, and other viruses in the home, city, airport immigration counter or check-in, hospitals, and busy clinics in a few seconds with the cost-effective point-of-care (POC) technology. Saliva collection is quite comfortable for patients as well as being easy, cheap, and non-invasive with minimal equipment required. It should also minimize the nosocomial transmission of 2019-nCoV to healthcare workers. Right now, in this controllable pandemic situation, all research centers, health agencies, and health care providers must explore the diagnostics opportunity and rapidly develop automated molecular point-of-care assays. This write-up will help epidemiologists, virologists, and clinicians to understand the importance of saliva in diagnostic testing as well as the transmission of the disease.
